# *Akkermansia muciniphila* Improves Depressive-Like Symptoms by Modulating the Level of 5-HT Neurotransmitters in the Gut and Brain of Mice

**DOI:** 10.1007/s12035-023-03602-6

**Published:** 2023-09-05

**Authors:** Huijuan Guo, Xinxu Liu, Ti Chen, Xiaoping Wang, Xiaojie Zhang

**Affiliations:** 1 Department of Psychiatry, National Clinical Research Center for Mental Disorders, National Center for Mental Disorders, The Second Xiangya Hospital of Central South University, No. 139, Renmin Middle Road, Furong District, Changsha, 410011 Hunan Province China; 2 Clinical Laboratory, The Third Xiangya Hospital, Central South University, Changsha, 410000 Hunan China

**Keywords:** *Akkermansia muciniphila* (AKK), Gut microbiome, 5-HT neurotransmitter, Chronic alcohol exposure, Chronic unpredictable mild stress (CUMS), Depression

## Abstract

Accumulating evidence has suggested that the gut microbiome plays an important role in depression. *Akkermansia muciniphila* (AKK), a next-generation probiotic, shows a beneficial effect on immune and metabolic homeostasis. The relative abundance of AKK was found negatively correlated with depressive symptoms in both clinical and pre-clinical studies. To evaluate the potential antidepressant effect of AKK and explore the possible mechanism, we used chronic alcohol exposure and chronic unpredictable mild stress (CUMS) to induce depressive-like behaviors in mice. We found that oral AKK administration significantly reduced the immobility time in the force swimming test (FST) and tail suspension test (TST) in the mice with chronic alcohol exposure and the CUMS mice. The sucrose preference in the mice receiving AKK was significantly increased in the sucrose preference test (SPT). More importantly, AKK implantation significantly increased the level of 5-HT in the gut and PFC of both the alcohol exposure mice and the CUMS mice. Furthermore, AKK had inhibited the expression of SERT in the gut but not in the brain for both NIAAA and the CUMS model mice. Interestingly, the expression of cFos in enteric nerves in the gut significantly decreased after AKK administration. In conclusion, our study demonstrated the antidepressant effect of AKK in mice exposed to alcohol exposure and CUMS, with the potential mechanism that AKK implantation might lead to an increased level of 5-HT and inhibited SERT expression in the gut, and might alter the gut-to-brain signal through suppression of enteric nerves activation.

**Supplementary Information:**

The online version contains supplementary material available at 10.1007/s12035-023-03602-6.

## Introduction

Depressive disorder is a common emotional disorder [1]. It is reported that nearly 300 million people experienced depressive disorder worldwide, and depression-related suicide have been estimated approximately 800,000 deaths every year. In addition, depression also has been ranked first of mental disorder disability-adjusted life-years (DALYs) [2, 3]. Currently, the treatment of depression mainly includes pharmacotherapy, psychotherapy, and physical therapy. Although most of the first-line antidepressants are safe and effective [4], there are still problems such as delayed effects, low response rate, and side reactions [5, 6]. Prior studies also showed that, among those who received first-line antidepressants, only 30–50% of the patients experienced relief of depressive symptoms [7–9]. Therefore, it is necessary to develop new adjunctive or synergistic treatments for depression that is safer and more effective with fewer side effects, to help improve the clinical outcome of patients with depression and reduce the burden of depression.

Alcohol abuse and stress are two of the major risk factors of depressive disorder. Studies found that the comorbidity of alcohol use disorder and depression was high [10, 11], as well as alcohol exposure could increase the risk of depressive disorder [12–14]. Previous studies demonstrated that patients with alcohol abuse have gut microbiome dysbiosis; this leads to increased gut permeability, which is positively correlated with persistent depression, anxiety, and craving after alcohol withdrawal [15, 16]. Furthermore, prior studies also found that stress was closely associated with depression and that stressful life events could induce a series of psychological and physiological changes [17]. In pre-clinical studies, alcohol exposure and stress stimuli are often used to induce typical depressive-like behaviors in rodents; thus, they are useful methods to investigate the neurobiology of psychological stress as well as depression and anxiety [13, 18, 19].

It is known that the bidirectional communication between gut microbiome and the brain is accomplished via the microbiome-gut-brain axis (MGB), which is primarily achieved through neuroendocrine and neuroimmune mechanisms involving the autonomic nervous system and the enteric nervous system. The CNS regulates gastrointestinal function and the enteric nervous system through the sympathetic and parasympathetic branches of the autonomic nervous system and the HPA axis. Therefore, it is speculated that the gastrointestinal system may influence regulatory effects through the transmission of intestinal metabolites, neuroactive compounds, and hormones via pathways encompassing the enteric nervous system, the vagus nerve, the circulatory system, and their interactions with the central nervous system [20, 21]. The gut microbiome in patients with depressive disorder was found significantly altered and changes in gut microbiota could also have implications for the function of the central nervous system and the emotional responses of patients [22, 23]. It has been found that the abundance and diversity of gut microbiota in patients with depression were significantly lower than those in healthy controls [24]. Yao et al. found that decreased abundance of Bacteroidales, Verrucomicrobiae, Bacteroidetes, Bacteroidia S24-7, and Bacteroides caccae was associated with the increase of depressive-like behaviors [14].

Transplantation of feces from depressed patients into rats can cause obvious depression-like behaviors such as anhedonia [24]. It has been found that some probiotics can be used as an adjunctive therapy for the treatment of anxiety and depression [25]. In animal studies, *Bifidobacterium* can exert an antidepressant effect by reducing the concentration of 5-hydroxyhonoacetic acid (5-HIAA) and dihydroxyphenylacetic acid (DOPAC) in the central nervous system of rats [26]. Supplementation with *Bifidobacteria* can also prevent depressive-like behaviors of mice induced by chronic stress to a certain extent [27, 28]. Sun et al. found that oral administration of *Clostridium* butyrate could promote the secretion of BDNF and significantly improve depressive-like behaviors of mice induced by chronic unpredictable mild stimulation (CUMS) [29]. In a clinical study, Wang et al. found that probiotic therapies could effectively improve the depressive symptoms and the concomitant gastrointestinal distress in patients with major depressive disorder [30]. Taken together, all of these findings indicate that gut microbiome is one of the factors and novel therapeutic targets for depressive disorders [31, 32].

*Akkermansia muciniphila* (AKK) is a gram-negative anaerobic bacterium that colonizes the mucus layer of the gut mucosa and accounts for 1–5% of the human gut microorganisms [33]. It is also considered as one of the next-generation probiotics. It has been found that AKK can help improve the gut mucosal barrier, reduce systemic inflammation, and improve glucose tolerance and insulin resistance; thus, it has broad prospects in the treatment of metabolic disorders and the maintenance of metabolic balance in the body [34–36]. Some studies suggest that AKK could improve the liver dysfunction induced by alcohol and has therapeutic potential in the management of depression. The relative abundance of AKK was found to be negatively correlated with depression [37]. Studies also found decreased relative abundance of AKK in patients with depression, alcohol addiction, Alzheimer’s disease, and cognitive impairments [38–40]. In addition, according to prior studies, AKK had little impact on the human body and almost no interaction with other medications. Nevertheless, the antidepressant role of AKK and the underlying mechanism of its antidepressant effect still remain largely unknown. Therefore, the present study aims to explore the antidepressant role of AKK and the possible mechanisms using different mouse models of depression.

## Materials and Methods

### Animals

Six- and eight-week-old male C57BL/6J SPF mice were obtained from Hunan Slack Animal Company, Changsha, China. The mice were raised in constant temperature and humidity conditions (temperature 22 ± 2 °C, humidity 55 ± 10%) with a stable light/dark cycle (12:12), with unlimited access to food and water. The protocol of the study was approved by the Animal Care and Use Committee of the Second Xiangya Hospital of Central South University and was in accordance with the institution’s guidelines for the care and use of laboratory animals.

### Culture of *Akkermansia muciniphila*

*Akkermansia muciniphila* (ATTC BAA-835) was cultured in BHI broth at 37 °C under strict anaerobic conditions (85% N_2_, 10%H_2_, and 5% CO_2_). Cultures were washed using PBS and concentrated in 25% (vol/vol) glycerol and then frozen and stored at – 80 °C. A representative glycerol stock was used to determine the colony-forming unit per milliliter (CFU/ml) by counting. Glycerol stocks were thawed and diluted to an end concentration of 2.5 × 10^9^ CFU/200 μl and 5% glycerol before administration by oral gavage.

## Experiment 1

- Step 1: establishment of mouse models of alcohol-induced depressive-like behavior—the National Institute on Alcohol Abuse and Alcoholism (NIAAA) model and the chronic alcohol gavage model

Both the NIAAA model and the chronic alcohol gavage model have been widely used in depression-related studies as they exhibit depressive-like behaviors [41]. The NIAAA model was established according to the study of Gao et al. [42]. In the present study, a total of 48 eight-week-old male mice were used to establish the NIAAA model (control diet group: *n* = 12; alcohol diet group: *n* = 12) and the chronic alcohol gavage model (alcohol + AKK group: *n* = 12; alcohol + glycerol group: *n* = 12). The animal experiment commenced after 1 week of adaptive feeding. After modeling, the comprehensive behavioral assessments, including the global locomotor abilities, depressive-like behavior, anxiety, or fear memory, were performed to ensure the successful construction of the mouse model of alcohol-induced depressive-like behaviors. More details are included in the Supplementary information: Supplementary Methods.

- Step 2: the schedule of AKK treatment on the NIAAA model and the chronic alcohol gavage model mice

**Fig. 1:**
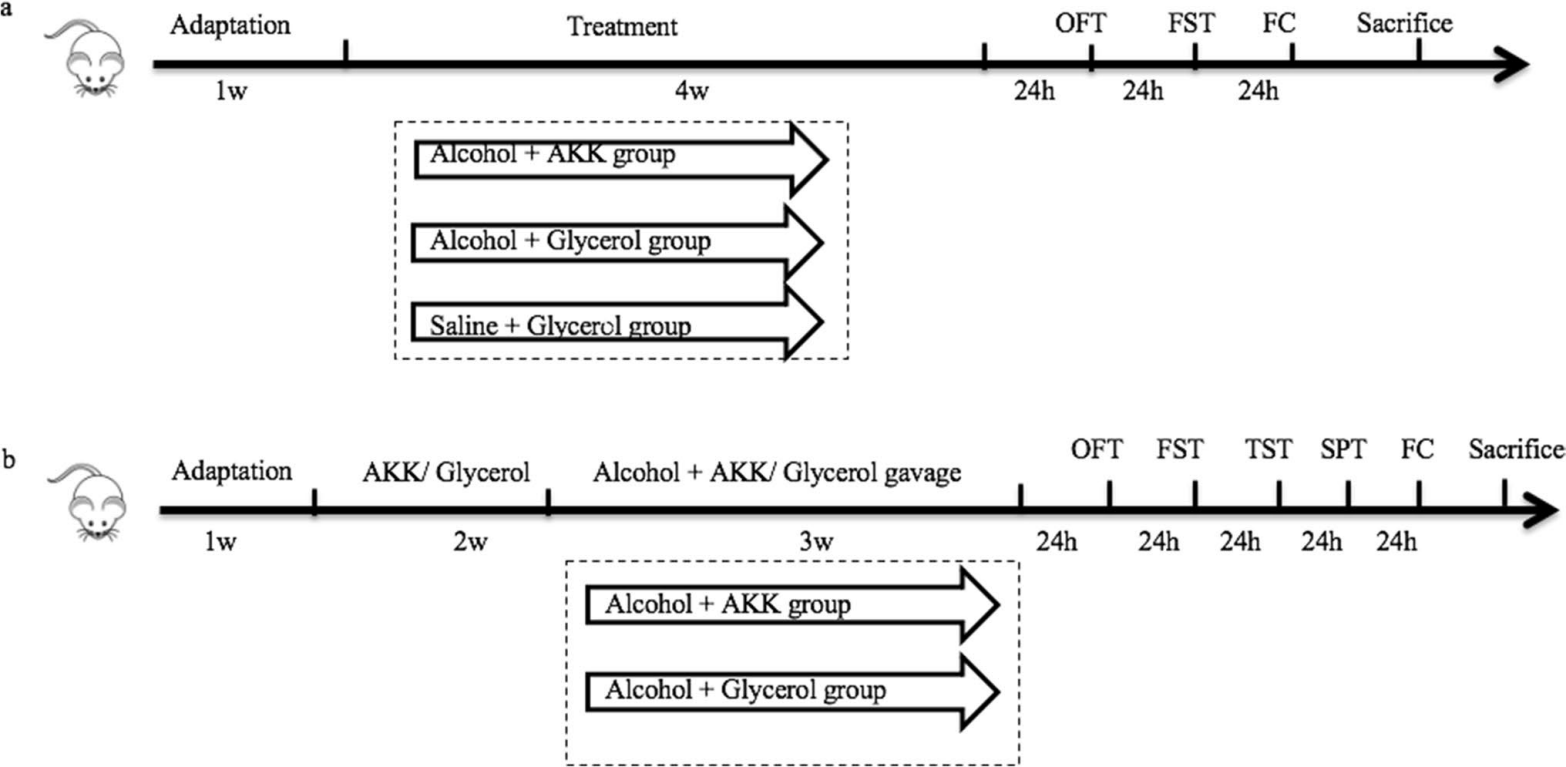
The procedure of AKK treatment for the chronic alcohol exposure mice. **a** The timeline of AKK treatment for NIAAA mice. **b** The timeline of AKK treatment for mice with chronic alcohol gavage

In the present study, the 36 mice aged 8 weeks were used to establish the NIAAA model of AKK intervention (alcohol + AKK group: *n* = 12; alcohol + glycerol group: *n* = 12; saline + glycerol group: *n* = 12). Other 24 mice of the same age were used to establish the chronic alcohol gavage model with AKK intervention (alcohol + AKK gavage group: *n* = 12; alcohol + glycerol gavage group: *n* = 12). A serious of behavioral parameters were used to evaluate the effect of AKK on alcohol-induced depressive-like behaviors in mice. The specific AKK treatment procedure for the NIAAA mice and the mice receiving chronic alcohol gavage are presented as follows:

All the NIAAA mice were given the Lieber-DeCarli alcohol liquid diet for 2 weeks after 1 week of adaptive feeding. The mice in the alcohol +AKK group were given AKK (2.5 × 10^9^ CFU/200 µl) in 5% glycerol by gavage once a day from the week 3, while the mice in the alcohol + glycerol group were given the same volume of 5% glycerol. From the 5th week, all mice were subjected to behavioral evaluation, including the open field test, forced swimming test, and conditional fear test. The two groups of mice were given a single dose of alcohol (20%, 5 g/kg) by gavage after the last behavioral test, and all the mice were euthanized 9 h after the last gavage. The specimens were collected and stored properly for further analyses. The mice were monitored for body weight on a weekly basis and food intake on a daily basis during the whole experiment (Fig. 1a). For the chronic alcohol gavage model, after 1 week of adaptive feeding, the mice of the two groups were pretreated with AKK (2.5 × 10^9^ CFU/200 µl) in 5% glycerol or the same volume of 5% glycerol via gavage, once a day for a total of 2 weeks. From the 3rd week, the two groups of mice pretreated with AKK/glycerol were given alcohol with gradually increased concentration, once a day for 3 weeks (the specific process was the same as that in step 1 of this experiment). AKK/glycerol was continuously administered during alcohol gavage, with AKK/glycerin administered by gavage at 9:00 a.m. and alcohol administered at 5:00 p.m. every day. The body weight and food intake levels were monitored on a daily basis. The behavioral tests, including the open field test, forced swimming test, tail suspension test, sucrose preference test, and conditional fear test, were started in the 6th week of the experiment at 2 h after the daily AKK gavage. After completing the behavioral tests, all the mice were euthanized 9 h after the last gavage. The specimens were collected and stored properly for further analyses (Fig. 1b).

### Experiment 2

- Step 1: Establishment of mouse model of depressive-like behavior induced by chronic unpredictable mild stress (CUMS).

A total of 45 6-week-old male C57BL/6J mice were used, and the experiment commenced after 2 weeks of adaptation. The mice were randomly divided into three groups: (1) control group (*n* = 15)—the blank control group, treated with glycerol gavage without CUMS to the end of experiment; (2) glycerol + CUMS group (*n* = 15)—the modeling control group, the mice were given the same volume of 5% glycerol via gavage for 5 weeks, and then exposed to CUMS for 4 weeks; (3) AKK + CUMS group (*n* = 15), treated with AKK (2.5 × 10^9^ CFU/200 µl) in 5% glycerol via gavage for 5 weeks, and then exposed to CUMS for 4 weeks. The mice were weighed on a weekly basis. The behavioral tests, including the open field test and the forced swimming test, were performed in the 12th week of the experiment. After completion of the behavioral tests, all the mice were euthanized 9 h after the last gavage. The specimens were collected and stored properly for further analysis. The detailed procedures are presented in the Supplementary information: Supplementary Methods.

The CUMS model has been widely used for studies of depression in recent years [43]. The depressive behaviors of animals after CUMS modeling are similar to the clinical manifestations of depression in humans induced by long-term exposure to stress; thus, this model can be used to simulate human depressive symptoms and a series of biochemical changes after depression [43]. The specific experimental procedures are formulated on the basis of earlier studies with some modifications [44–46]. More details of the procedures are included in the Supplementary information: Supplementary Methods.

- Step 2: The schedule of AKK administration on the CUMS mice

After adaptive feeding, the mice in the AKK + CUMS group were pretreated with AKK (2.5 × 10^9^ CFU/200 µl) in 5% glycerol, and the mice in the control and the glycerol + CUMS groups were given the same volume of 5% glycerol; all mice were treated once a day for a total of 5 weeks. AKK/glycerol was continuously administered during the CUMS modeling and behavioral tests.

### Behavioral tests

The details of the behavioral tests are presented in the Supplementary information: Supplementary Methods.

### Collection of Serum, Brain, and Intestinal Tissues

The mice were euthanized after the experiment. Before that, pericardial blood specimens were quickly gathered from the anesthetized mice and placed in anticoagulation tubes, which were then kept upright at room temperature for 30 min. The blood specimens were centrifuged at 3000 r/min for 10 min at 4 °C, and the supernatant was collected for analysis. An automatic biochemical analyzer was used to measure the levels of alanine aminotransferase (ALT) and aspartate aminotransferase (AST) for the evaluation of the mice’s liver function. Tissues from the prefrontal cortex (PFC) and the gut were collected and stored in a freezer at – 80 °C.

### Analysis of 5-HT in the Gut and Brain Tissues

Before analysis, the brain tissue was thawed on ice and weighed, and freshly prepared homogenate (H3ClO4 0.1 M, EDTA Na2 0.1 mM, Internal standard DHBA) was added. The tissue was then homogenized at – 40 °C (2 mm zirconia beads, 60 HZ, 60 s, once, Beijing Hede Technology Co., Ltd., Jing N-9548R). The small intestine tissue was collected and the mesentery was removed from it. The tissue was then washed 3 times with prechilled normal saline; after dried with filter paper and weighed, the tissue was added with freshly prepared homogenate at a dose of 5 µl per mg tissue sample (H3ClO4 0.2 M, EDTA Na2 0.1 mM, internal standard DHBA) and homogenized at – 40 °C (2 mm zirconia beads, 60 HZ, 30 s, with a space of 5 s, repeated for 3 times, Beijing Hede Technology Co., Ltd., Beijing N-9548R). Then, both samples were centrifuged at low temperature (20,000 rpm, 30 min, 4 °C), and the supernatant was collected for further analysis. The levels of 5-HT in the supernatant of mouse gut tissue homogenate and the supernatant of brain tissue homogenate were measured using the ESA-HPLC-ECD Kit and the commercial 5-HT ELISA Kit, respectively.

### RNA Extraction and qPCR

The total RNA was isolated from the brain or intestine tissues using the Trizol reagent. The ThermoscripTM RT-PCR system (Promega) was used to synthesize the first strand cDNA using 1 μg of the total RNA as template according to the manufacturer’s instructions. The newly synthesized cDNA templates were amplified by DreamTaq Green PCR Master Mix (2×) (Fermentas) in a 20 µl reaction volume using Ligtcycler480 (Roche). For the qPCR, the mTPH1 forward primer was AAGAAATTGGCCTGGCTTC, the mTPH1 reverse primer was GTTTGCACAGCCCAAACTC, the mSERT forward primer was TGGGCGCTCTACTACCTCAT, the mSERT reverse primer was ATGTTGTCCTGGGCGAAGTA, the mcFos forward primer was TTTCAACGCCGACTACGAGG, and the mcFos reverse primer was TCTGCGCAAAAGTCCTGTGT.

### Statistics

Statistical analysis was performed using the GraphPad Prism 6.0 software. All data were presented as mean ± standard error (mean ± SEM). The data were tested for normality using the Shapiro-Wilk method, and the homogeneity of variance was analyzed using the *F* test. The significance testing between two groups was performed using the unpaired two-samples Student’s or Welch’s *t* test, and comparison between multiple groups was conducted using one-way analysis of variance (ANOVA), with *P* < 0.05 indicating that the difference was statistically significant.

## Results

### Experiment 1

#### The Chronic Alcohol Exposure Slowed Weight Gain and Induced Depressive-Like Behavior in Mice

**Fig. 2:**
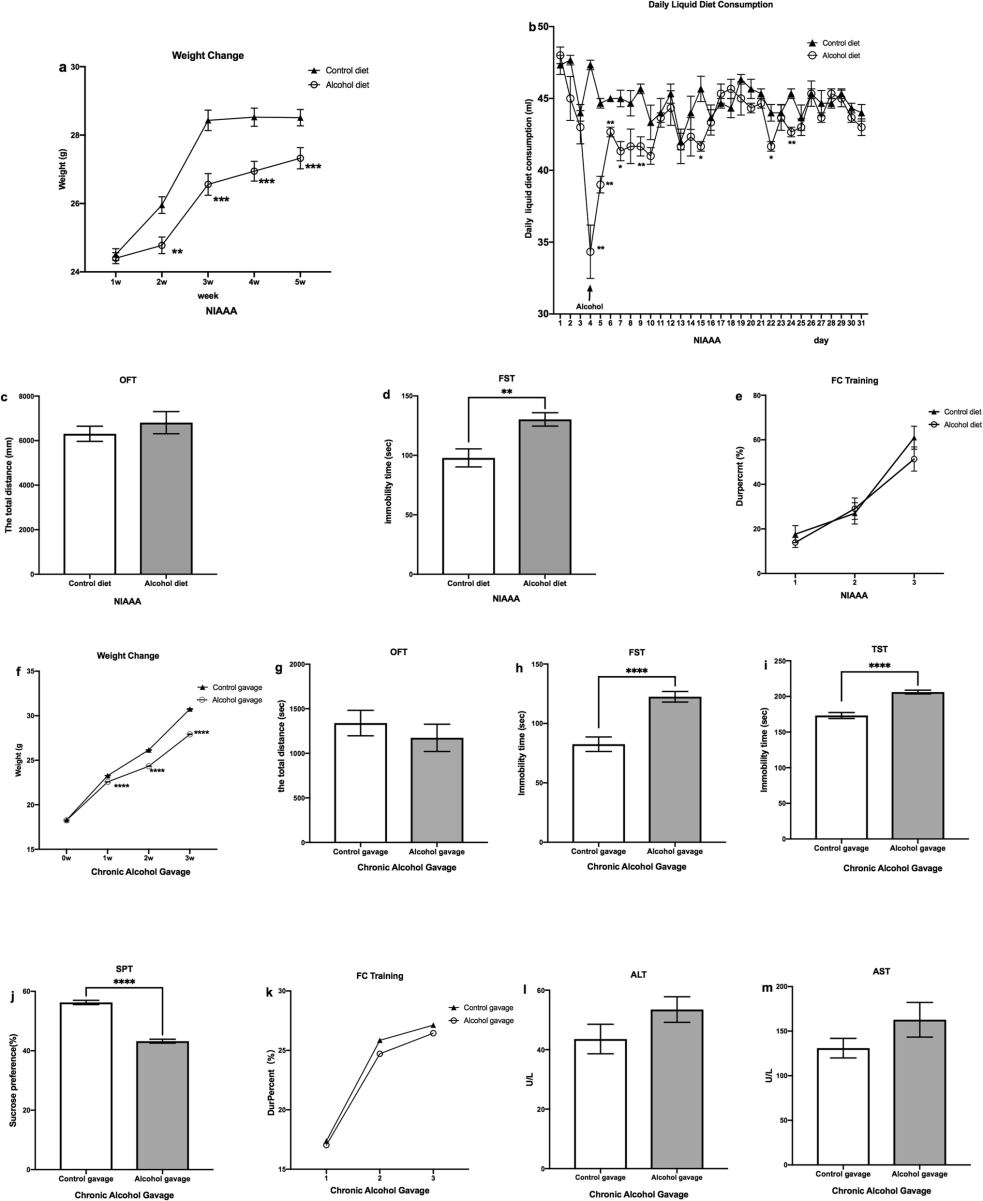
The alcohol exposure slowed weight gain and induced depressive-like behaviors in mice. **a** The weight change of NIAAA mice. **b** The daily liquid diet consumption of NIAAA mice. **c** Total distance covered by NIAAA mice in OFT. **d** Immobility time of NIAAA mice in FST. **e** The Dur-percent (%) in FC training in NIAAA mice. **f** The weight change of mice with chronic alcohol gavage. **g** Total distance covered by mice with chronic alcohol gavage in OFT. **h** Immobility time of mice with chronic alcohol gavage in FST. **i** Immobility time of mice with chronic alcohol gavage in TST. **j** Immobility time of mice with chronic alcohol gavage in SPT. **k** The Dur-percent (%) in FC training in mice with chronic alcohol gavage. **l** The ALT level of mice with chronic alcohol gavage. **m** The AST level of mice with chronic alcohol gavage. Data are presented as means ± SEM; *, **, and *** indicate *P* < 0.05, *P* < 0.01, and *P* < 0.001, respectively

Similar to previous studies [47], the weight of the mice with alcohol exposure (including alcohol diet and alcohol gavage) was significantly lower than that of the control groups (*P* < 0.01, Fig. 2a, f). Mice in the alcohol diet group had significantly less daily liquid diet consumption than the control diet group (*P* < 0.001, Fig. 2b). After chronic alcohol exposure, the mice showed depression-like behaviors, which mainly manifested as prolonged immobility time in the FST for the NIAAA mice given alcohol diet (*P* < 0.01, Fig. 2d); compared with the control gavage group, the mice treated with alcohol gavage had significantly prolonged immobility time in the FST (*P* < 0.0001, Fig. 2h) and TST (*P* < 0.0001, Fig. 2i); the sucrose preference of the alcohol gavage group was also significantly decreased in the SPT (*P* < 0.0001, Fig. 2j). In the present study, we also found that there was no difference in the total distance of movement in the OFT (*P* > 0.05, Fig. 2c, g) and in the freezing time Dur-percent (%) (*P* > 0.05, Fig. 2e, k) in the FC training test between the NIAAA mice given alcohol diet/the alcohol gavage group and the control groups. In addition, the levels of ALT and AST of the alcohol gavage group were higher than those of the control gavage group (*P* > 0.05, Fig. 2l, m), although the differences were not significant; this also indicated that alcohol gavage could induce liver damage in mice. These above results demonstrated that chronic alcohol exposure could induce depressive-like behaviors in mice but with little impact on their overall spontaneous movements and fear memory.

#### AKK Alleviated Depressive-Like Behaviors of Both the NIAAA Mice and the Mice Treated with Alcohol Gavage

**Fig. 3:**
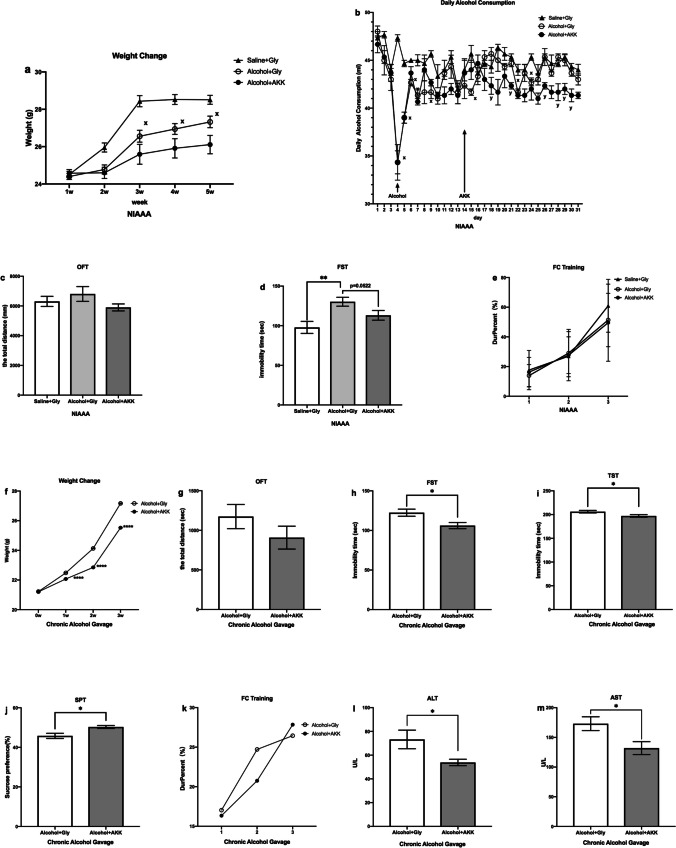
AKK ameliorates depression-like behavior in chronic alcohol exposure mice. **a** The change of weight of NIAAA mice. **b** The daily liquid diet consumption of NIAAA mice. **c** Total distance covered by mice in OFT of NIAAA mice. **d** Measurement of immobility time in FST of NIAAA mice. **e** The Dur-percent (%) in FC training of NIAAA mice. **f** The weight change of Chronic Alcohol Gavage mice. **g** Total distance covered by mice in OFT of chronic alcohol gavage mice. **h** Measurement of immobility time in FST of chronic alcohol gavage mice. **i** Measurement of immobility time in TST of chronic alcohol gavage mice. **j** The sucrose preference in the SPT of chronic alcohol gavage mice. **k** The Dur-percent (%) in FC training of chronic alcohol gavage mice. **h** The level of ALT of chronic alcohol gavage mice. **i** The level of AST of chronic alcohol gavage mice. Data are presented as means ± SEM and *n* = 7–12/group, ^x^*P* < 0.05 for the alcohol + glycerol group vs. the saline + glycerol group; ^y^*P* < 0.05 for the alcohol + AKK vs. the alcohol + glycerol group, * and *** indicate *P* < 0.05, *P* < 0.01, respectively

In the present study, the results showed that in the NIAAA model the weight of the mice in the alcohol + AKK group was lower than that in the alcohol + glycerol group and the saline + glycerol group (Fig. 3a). In the chronic alcohol gavage model mice, the weight of the mice in the alcohol + AKK group was also lower than that in the alcohol + glycerol group after AKK administration (*P* < 0.05, Fig. 3f), which was in line with previously reports [34]. With regard to the NIAAA model, the alcohol exposure mice exhibited longer immobility time in the FST (*P* < 0.01, Fig. 3d), which showed a reducing trend after AKK intervention (*P* = 0.0522, Fig. 3d); but there was no difference in the total movement distance in the OFT (*P* > 0.05, Fig. 3c) and the Dur-percent (%) in the FC training (*P* > 0.05, Fig. 3e) between the three groups of mice. For the mice treated with chronic alcohol gavage, the group treated with AKK had significantly decreased the immobility time in the FST (*P* <0.05, Fig. 3h) and TST (*P* < 0.05, Fig. 3i), as compared with the group treated with glycerol gavage; the sucrose preference in the group receiving AKK was significantly increased in the SPT (*P* < 0.05, Fig. 3j); For other behavioral tests, the AKK and glycerol treatments showed little difference in the total movement distance in the OFT (*P* > 0.05, Fig. 3g) and the Dur-percent (%) in the FC training (*P* > 0.05, Fig. 3k) between the two groups. The above results suggested that AKK could effectively alleviate depressive-like behaviors induced by chronic alcohol exposure with little impact on the spontaneous activity and fear memory of mice. Furthermore, we found that the ALT and AST levels in the chronic alcohol gavage model mice with AKK treatment were significantly lower than those with glycerol treatment (*P* < 0.05, Fig. 3l, m), indicating that AKK could effectively improve the liver injury caused by alcohol gavage, which was consistent with previous findings [48].

#### AKK Regulated the Level of 5-HT Serotonergic Neurotransmitters in the Gut and Prefrontal Cortex (PFC) of the NIAAA Mice and the Chronic Alcohol Gavage Model Mice

**Fig. 4:**
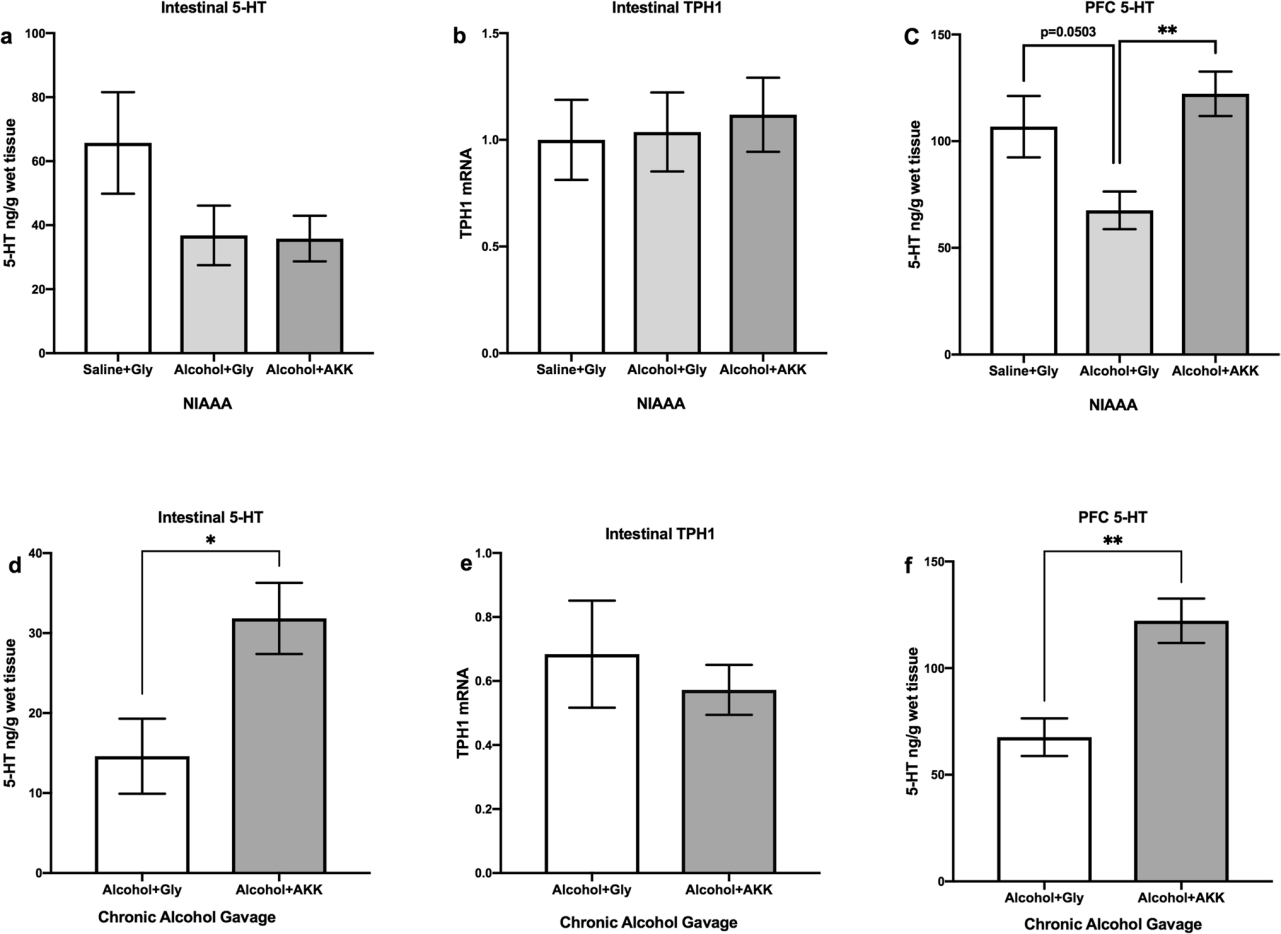
AKK ameliorated the level of 5-HT serotonergic neurotransmitters in mice with chronic alcohol exposure. **a** The 5-HT concentration in the intestine of NIAAA mice. **b** The TPH1 level in the intestine of NIAAA mice. **c** The 5-HT concentration in the PFC of NIAAA mice. **d** The 5-HT concentration in the intestine of chronic alcohol gavage mice. **e** The TPH1 level in the intestine of chronic alcohol gavage mice. **f** 5-HT concentration in the PFC of chronic alcohol gavage mice. Data are presented as means ± SEM; * and ** indicate *P* < 0.05 and *P* < 0.01, respectively

In this study, we found that the levels of serotonergic neurotransmitters could be regulated by AKK treatment for both the NIAAA mice and the chronic alcohol gavage model mice. Specifically, the results showed that there was no significant difference in the level of 5-HT in the gut or PFC between mice of alcohol + glycerol and the saline + glycerol group (*P* > 0.05, Fig. 4a, c) in NIAAA experiment; and AKK intervention had increased the 5-HT levels in the gut of the chronic alcohol gavage model mice (*P* < 0.05, Fig. 4d), but did not significantly affect the 5-HT levels in the gut of the NIAAA mice (*P* > 0.05, Fig. 4a). For both the NIAAA model and the chronic alcohol gavage model, compared to glycerol intervention, the intervention with AKK was associated with increased levels of 5-HT in the PFC (*P* < 0.01, Fig. 4c, f) in the mice with alcohol exposure. Furthermore, we found that there was no significant difference of the expression of TPH1, the rate-limiting enzyme of 5-HT synthesis in enteric endocrine cells (EECs) between the mice treated with AKK and the mice treated with glycerol (*P* > 0.05, Fig. 4b, e). These above results indicated that AKK could increase the levels of 5-HT both in the gut and in the brain; however, the increased 5-HT in the gut might not be related to the activation of 5-HT synthesis in EECs.

### Experiment 2

#### AKK Ameliorated CUMS-Induced Depressive-Like Behaviors in Mice

**Fig. 5:**
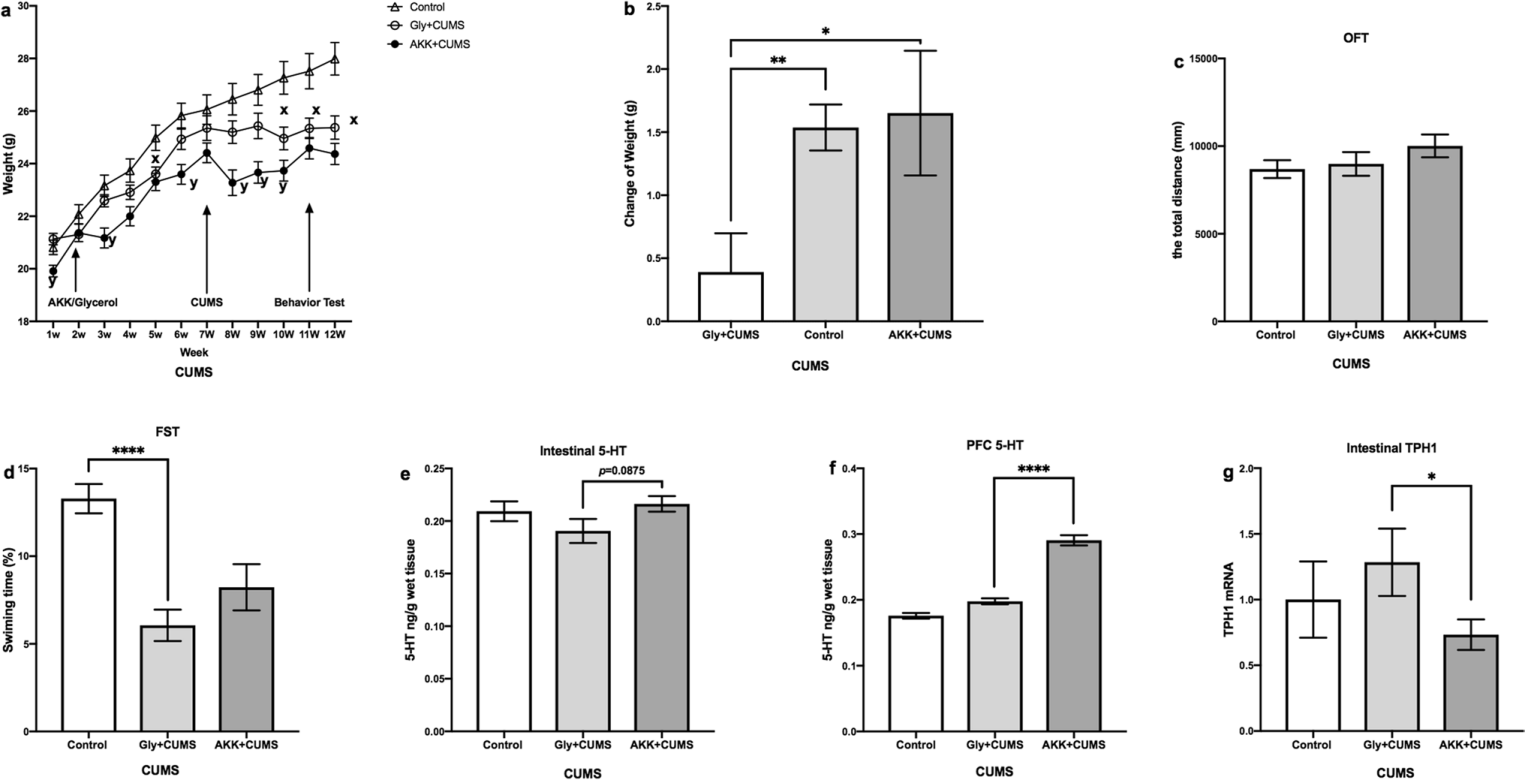
AKK ameliorated CUMS-induced depressive-like behaviors and the level of 5-HT in mice. **a**–**b** The change of weight. **c** Total distance covered by mice in OFT. **d** Swimming time in FST. **e** The 5-HT concentration in the intestine. **f** The 5-HT concentration in the PFC. **g** The TPH1 concentration in the intestine. Data are presented as means ± SEM and *n* = 13–15/group. ^**x**^*P* < 0.05 for the glycerol + CUMS group vs. the control group; ^**y**^*P* < 0.05 for the AKK + CUMS group vs. the glycerol + CUMS group. * and **** indicate *P* < 0.05 and *P* < 0.0001, respectively

To further investigate the antidepressant effect of AKK, a CUMS-induced depressive mouse model was used in this study. As shown in Fig. 5a, the weight of the mice in the control group continued to increase. Compared with the control group, the mice of the glycerol + CUMS group stopped gaining weight by the end of the modeling. The mice of the AKK + CUMS group showed a significant weight loss in the first week of modeling, but in the 8th week, there was a tendency of increase similar to the control group; however, the overall body weight of those treated with AKK + CUMS was still lower than the other two groups. The results showed that AKK had decreased the weight of mice (Fig. 5a), and the weight change in mice of glycerol + CUMS was lower than that of the control (*P* < 0.01, Fig. 5b) and the AKK + CUMS groups (*P* < 0.05, Fig. 5b). In the OFT, there was no difference in the total distance between the three groups of mice (*P* > 0.05, Fig. 5c). The CUMS mice exhibited shorter swimming time in the FST (*P* < 0.0001, Fig. 5d), which showed an increasing trend after administration of AKK, although the increase was not statistically significant (*P* = 0.197, Fig. 5d). These findings indicated that the modeling of CUMS-induced depression was successful and AKK may have the potential to ameliorate CUMS-induced depression-like behavior in mice.

#### AKK Increased 5-HT Levels in the Gut and Prefrontal Cortex (PFC) of CUMS Mice

We also analyzed the 5-HT levels in the gut and brain of these CUMS mice, which showed no significant difference in the level of 5-HT in the gut between mice of glycerol + CUMS and the control group (*P* >0.05, Fig. 5e) as well as an increasing trend in the level of 5-HT during chronic stress treatment after intervention with AKK (p = 0.0875, Fig. 5e); in addition, AKK intervention also inhibited the expression of TPH1 (*P* < 0.05; Fig. 5g). We also found that there was no significant difference in the levels of 5-HT in the PFC between the mice of glycerol + CUMS and the control group (*P* > 0.05, Fig. 5f), but AKK treatment significantly had increased the levels of 5-HT of the CUMS mice (*P* < 0.001, Fig. 5f).

### AKK Suppressed the SERT in the Gut and the Enteric Nerve Activation

**Fig. 6:**
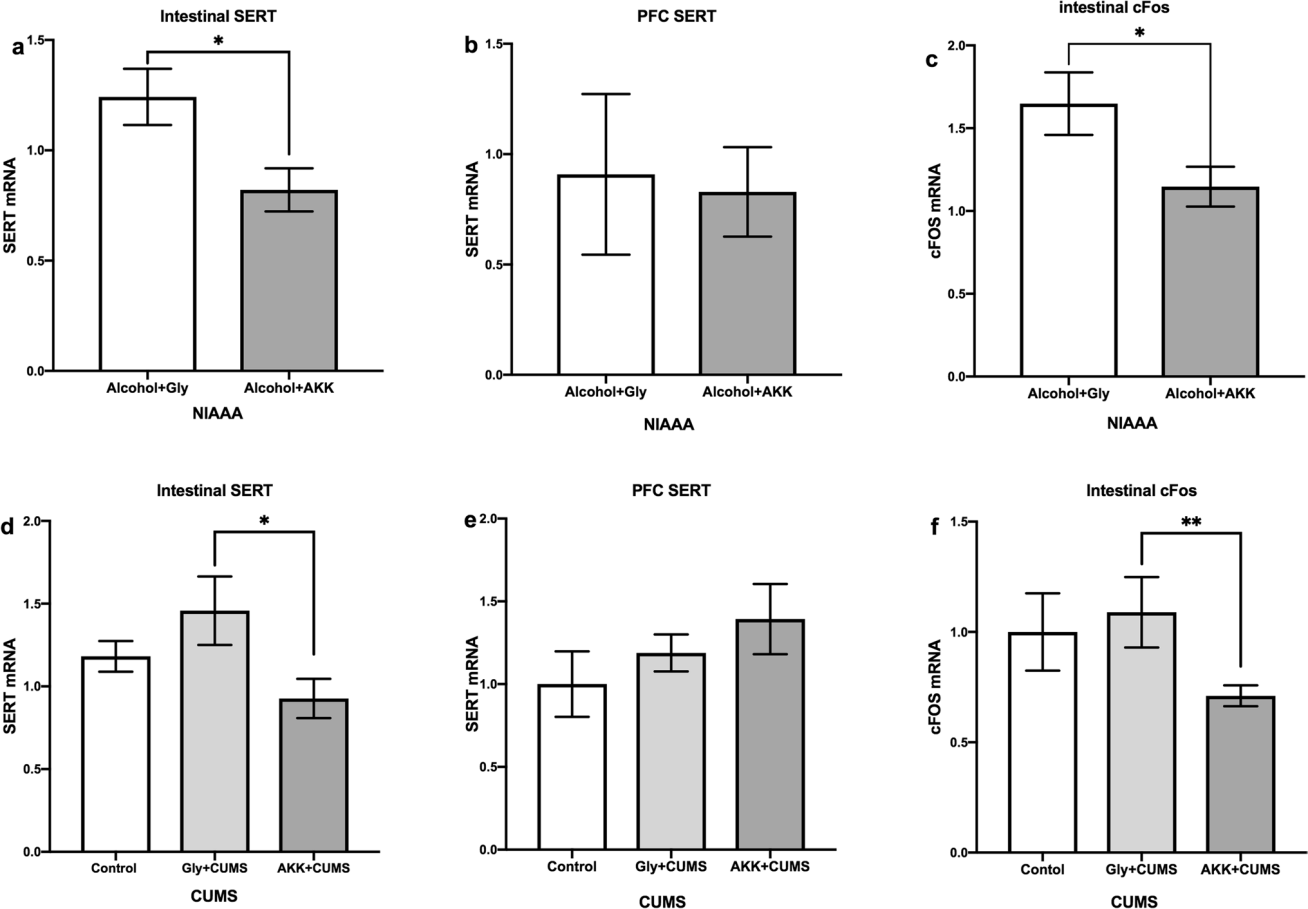
AKK suppressed the SERT in the gut and the expression of cFos in enteric nerves. **a** The SERT level in the intestine in NIAAA mice. **b** The SERT level in the PFC in NIAAA mice. **c** The cFos level in enteric nerves in NIAAA mice. **d** The SERT level in the intestine in CUMS mice. **e** The SERT level in the PFC in CUMS mice. **f** The cFos level in enteric nerves in CUMS mice. Data are presented as means ± SEM; * and ** indicate *P* < 0.05 and *P* < 0.01, respectively

It is interesting that AKK inhibited the expression of serotonin transporter (SERT) in the gut (*P* < 0.05, Fig. 6a, d), but no effect was found on the expression of SERT in the brain (*P* > 0.05, Fig. 6b, e) in both the chronic alcohol exposure model and the CUMS model. These findings indicated that the increasing levels of 5-HT might have resulted from the inhibition of SERT and that the suppression of SERT expression in the gut might have altered the activation of enteric nerves. Thus, we also analyzed the cFos level in the gut, which showed that the expression of cFos in enteric nerves was significantly decreased after AKK administration in the mice of both the chronic alcohol exposure model (*P* < 0.05, Fig. 6c) and the CUMS model (*P* < 0.01, Fig. 6f). These findings indicated that AKK might have altered the gut-to-brain signal through suppression of enteric nerve activation.

## Discussion

In the present study, mice with depressive-like behaviors induced by alcohol exposure or CUMS were used as animal models to explore the effect of AKK intervention on depressive-like behaviors of mice and the possible mechanism. The results showed that AKK intervention could improve the depressive symptoms and increased the levels of 5-HT serotonergic neurotransmitters in the gut and brain of mice.

In the study, we used three behavioral tests, i.e., the forced swimming test, sucrose preference test, and tail suspension test, to assess depressive-like behaviors of mice, with the forced swimming test and tail suspension test used to measure the rodent’s depressive-like behaviors and the immobility time used to reflect behavioral despair [49, 50]. The sucrose preference test was used to assess anhedonia, a key symptom of depression in humans. Spontaneous activities and global locomotor abilities were assessed using the open field test [51]. After establishment of mouse models of depression, the mice treated with alcohol had significantly increased the immobility time in the FST and TST and had decreased sucrose preference in the SPT compared to those of the control group; the mice treated with CUMS exhibited shorter swimming time in the FST. The above results indicated that all the mouse models of depression were successfully established. Furthermore, AKK intervention could improve the depressive-like behaviors of both the NIAAA mice and the chronic alcohol gavage mice; for example, AKK intervention significantly reduced the immobility time in the FST of the NIAAA mice with alcohol exposure. Compared with the mice treated with glycerol gavage, those given AKK gavage had significantly decreased immobility time in the FST and TST; the sucrose preference in the group given AKK was significantly increased in the SPT. The swimming time showed an increasing trend after AKK treatment in the CUMS mice. Our results were in line with previous reports [37, 52]. Meanwhile, there was no significant difference between mice with AKK treatment and the control group in the total traveled distance in the open field test, and no significantly difference in freeze time in fear conditioning, all of the results suggested that the AKK intervention specific improved the depressive-like behaviors but had no significant effect on the global locomotor abilities, anxiety, or fear memory of the mice.

Additionally, we also found that AKK implantation significantly increased 5-HT levels in the gut and PFC of both the chronic alcohol exposure mice and the CUMS mice. These findings might be one of the important mechanisms of AKK alleviating the depressive-like behaviors in these mice models. 5-HT is one of the classic neurotransmitters associated with the pathophysiology of depression [53]. A lower level of 5-HT is often observed in patients with depression, and 5-HT deficiency has been hypothesized as one of the causes of depression since last century [54]. Approximately 90% of 5-HT in the human body exists in the gut pheochromocytoma, and about 2% exists in the central nervous system. It is converted from tryptophan (TRP) to 5-hydroxytryptophan (5-HTP) through TPH, and then synthesized in gut chromaffin cells and central neurons of the digestive tract through 5-hydroxytryptophan decarboxylase [55]. On the one hand, the intestinal 5-HT may play an antidepressant role by activating the enteric nerve and vagus nerve and then transmit it to the brain. On the other hand, the gut microbiome itself has the ability to participate in the tryptophan metabolic pathway, which may affect the central 5-HT synthesis by increasing the intermediate products in the metabolic pathway that can pass through the blood-brain barrier. Furthermore, the ability of oral SSRI drugs to pass through the blood-brain barrier is inconsistent [56]. The first barrier for these drugs to act in the body is the intestinal tract, where SERT is expressed in the intestinal nerves. Although there is insufficient evidence in this area, it also cannot be ruled out the antidepressant effects of SSRI drugs acting on the gut. However, the dysregulation of 5-HT system might not be the only neurobiological mechanisms for CUMS model and depressive-like behaviors. Here, we only focus on the AKK improved depressive-like symptoms caused by CUMS via 5-HT pathway, but we also noticed that AKK might also affect the other neurotransmitters pathway, as well as immune system. Thus, AKK improved the depressive-like symptoms caused by CUMS might be through comprehensive and combination mechanisms. It might be the scope of our future study.

In this study, we first speculated that AKK might activate the serotonin pathway by increasing the activity of TPH1 in the gut. However, in our study, we found that AKK did not affect the level of TPH1 in the gut of the NIAAA mice and the chronic alcohol-treated mice; in the CUMS mouse model, AKK intervention had decreased the expression of TPH1 in the gut. These results showed that the AKK increased 5-HT levels in the gut were independent of the expression of TPH1 in host gut cells.

Secondly, we found that AKK treatment had inhibited the expression of SERT in the gut but had no effect on the expression of SERT in the brain of both the NIAAA mice and the CUMS mice. SERT is highly expressed both in brain and gastrointestinal tract. The first-line antidepressant drugs (SSRIs, including fluoxetine and paroxetine, etc.) [57] selectively target to SERT and inhibit the reuptake of 5-HT in the central nervous system, indirectly increasing the concentration of 5-HT in the synaptic space and producing an antidepressant effect. Therefore, it is possible that decrease of SERT expression may be one of the reasons for the increase of 5-HT, and the mechanism by which AKK leads to a decrease of intestinal SERT worth further investigation.

Additionally, 5-HT could not cross the blood-brain barrier (BBB), but TRP and 5-HTP could cross the BBB using the canine amino transporter. 5-HT is mainly metabolized into 5-hydroxytryptamine indoleacetic acid (5-HIAA) through the liver, and the latter is excreted through the kidney. Whether 5-HT in the gut affect 5-HT synthesis in brain was also explored. In the present study, we found that the expression of cFos in enteric nerves was significantly decreased in both the NIAAA mice and the CUMS mice after AKK treatment, indicating that AKK might have altered the gut-to-brain signaling through enteric nerves. This result was consistent with the latest report that cFos in gut-extrinsic sympathetic neurons was increased in germ-free mice while suppressed by re-colonization of bacteria [58]. This indicated that the microbiota could modulate enteric nerves via the gut-brain circuit. The mechanism of AKK altering 5-HT levels in the gut and brain might be its modulation of the enteric nerves via the gut-brain circuit. However, according to a study of Paul et al., monocolonization of germ-free mice treated with AKK did not result in reduced levels of cFos [58]; in the present study, monocolonization of SPF mice treated with AKK did result in reduced cFos levels. Taken together, the different findings might indicate that the presence of AKK only was not enough to suppress the cFos level, and it was possible that the interaction between the bacterial consortia treated with AKK and other bacteria suppressed the enteric nerves.

There are still some limitations in this study. The interaction between AKK and other enterobacteria could not be ruled out. Also, we did not analyze global gut-derived metabolites; the effect of AKK on the improvement of depressive-like behaviors in mice might be the result of modulating on various gut-derived metabolites including other neurotransmitters. Additionally, here we only examined the mRNA level of TPH1; we try to test the protein level of TPH1 in intestine by Western blot but always failed. Finally, whether AKK could promote 5-HT synthesis and metabolism in the in vitro environment requires further investigation.

In conclusion, the present study found that AKK intervention had improved the depressive-like behaviors by affecting the levels of 5-HT in both mice with alcohol exposure and mice treated with CUMS. As one of the next-generation probiotics, AKK plays an active role in improving gut barrier function and regulating immune responses. This study is helpful for the further understanding the microbiota-gut-brain axis and has proposed new ideas for the study of the role and mechanism of AKK in depression and alcohol use disorder. Hopefully, our findings could provide a basis for the integration of the probiotic AKK into the comprehensive treatment of depression.

### Supplementary Information

Below is the link to the electronic supplementary material.

Supplementary file1 (DOCX 120 KB)

## Data Availability

All data generated or analyzed during this study are included in this published article.
