# Supplementary material for: Akkermansia muciniphila Improves Depressive-Like Symptoms by Modulating the Level of 5-HT Neurotransmitters in the Gut and Brain of Mice
Source: Mol Neurobiol. 2023 Sep 5;61(2):821–34. doi: 10.1007/s12035-023-03602-6 (PMC10861622; doi:10.1007/s12035-023-03602-6)
Supplement: Supplementary file 1 — Supplementary file1 (DOCX 120 KB) [file 12035_2023_3602_MOESM1_ESM.docx]

Supplemental information for

***Akkermansia muciniphila* improves depressive-like symptoms by modulating the level of 5-HT neurotransmitters in the gut and brain of mice**

Huijuan Guo^a^, Xinxu Liu^a^, Ti Chen^b^, Xiaoping Wang^a^, Xiaojie Zhang^a#^

**^#^Corresponding author**: Xiaojie Zhang, National Clinical Research Center for Mental Disorders, Department of Psychiatry, The Second Xiangya Hospital of Central South University, No. 139, Renmin Middle Road, Furong District, Changsha. **Email address:** [xiaojiezhang2014@csu.edu.cn](mailto:xiaojiezhang2014@csu.edu.cn)

The file includes:

Supplementary methods

Supplementary Figure 1

Supplementary Figure 2

**Supplementary methods**

**Experiment 1**

**Step 1: Establishment of mouse models of alcohol induced depressive-like behavior – the National Institute on Alcohol Abuse and Alcoholism (NIAAA) model and the chronic alcohol gavage model**

During the establishment of the NIAAA model(Supplementary Figure 1a), feeding method of the liquid forage with equal calories (the alcohol liquid and control liquid feed formulations were provided by Nantong Trophy Co., Ltd.) was adopted. Mice in the alcohol diet group were given Lieber-DeCarli alcohol liquid forage (standard type) for 4 weeks, followed by a free diet through the end of the experiment. Mice in the control diet group were given Lieber-DeCarli control liquid forage. From the 5th week, all the mice were subjected to a series of behavioral tests, including the open field test (OFT), forced swimming test (FST), and conditional fear test. The alcohol diet and the control diet were continued through the last day of the behavioral tests. Before the beginning of each behavioral test, the mice were starved for 2 hours in the behavior test room. After the last behavioral test, the mice in the alcohol diet group were given a single dose of alcohol (20%, 5 g/kg body weight)[1]by gavage, while the control diet group mice were given maltose gavage solution (9 g/kg body weight). The establishment of the chronic alcohol exposure model took 3 weeks in total(Supplementary Figure 1b). The mice in this group were given an initial dose of alcohol of 2 g/kg in week 1, once a day; the dose was then increased to 3 g/kg once daily in week 2, and 4 g/kg once daily in week 3. The behavioral tests were started in the 3rd week of the experiment. The alcohol was administered by gavage through the last day of the behavioral tests and after the behavioral tests at 5:00 pm every day. After completing the behavioral tests, the mice were starved overnight and sacrificed 30 minutes after a final alcohol gavage (20%, 5 g/kg).

**Experiment 2**

**Step 1: Establishment of mouse model of depressive-like behavior induced by chronic unpredictable mild stress (CUMS).**

The procedure of the CUMS modeling and the timeline of AKK administration for CUMS mice are presented in Supplementary Figure 2. The modeling of CUMS mice included 10 approaches to induce stress: food/water deprivation (24 h), tail clipping (1 min), heat stress (45°C, 5 min), swimming in cold water (5℃, 10 min), cage tilting 45° (24 h), light and dark inversion, wet bedding (10 h), behavioral restriction (1-2h), and electric shock to the sole of the foot (36V alternating current, stimulated once/1 min, with each stimulation lasting for 10 s, for a total of 30 times). The mice received 1-2 mild stressors randomly per day, with the same stressor not applied in two consecutive days. Each stressor should be administered for no more than 2-3 times cumulatively, and the intervention would last for 4 weeks. During the modeling period, glycerol and AKK viable bacteria were administered by gavage. During the experiment, one mouse in the glycerol + CUMS group died in the 8th week and one died in the 11th week; 2 mice in the AKK + CUMS group died in the 11th week. According to statistics, all the deaths occurred during the modeling period, when the mice were swimming in ice water. The numbers of mice that finally participated in behavioral data analyses and sampling were: n=15 for the Control group, n=13 for the glycerol + CUMS group, and n=13 for the AKK + CUMS group.

**Behavioral tests**

**The open field test (OFT):** This test is used to evaluate the autonomous behavior, spontaneous exploration behavior and the anxiety level of mice in a new environment[2,3]. All the mice to be tested were first placed in the behavioral laboratory for 2 hours for acclimatization to the environment. At the start of the test, the mice were carefully placed in the central area of the open-field experimental box, with timing and video recording started immediately. The total moving distance of the mice within 4 minutes and the residence time in the central area were automatically recorded. After the completion of each behavioral test, the mice were removed and their traces (urine, feces, odor, etc.) left in the open field experiment box were removed using clean and odor-free paper towels.

**The sucrose preference test (SPT):** The entire experiment lasted for 5 days. The mice were single-cage housed with two 200 ml water bottles that could be accurately metered and sealed. The mice were given two bottles of 1% sucrose solution on days 1-2 as well as a bottle of distilled water and a bottle of 1% sucrose solution on days 3-4; the position of the two bottles was altered for four or more times during the training. On the 5th day, the mice were given one bottle of distilled water and one bottle of 1% sucrose solution after fasting for 12 hours. The consumption of sucrose-sweetened water and distilled water within 2 hours was measured to calculate the sucrose preference index. The position of the two bottles was altered every half an hour during the measurement. At the start, the two bottles were positioned randomly for each animal. The sucrose preference rate reflects the preference of sweetened water in mice, and anhedonia reflected by decreased sucrose preference rate is one of the most important indicators of depression-like behaviors. The sucrose preference rate was calculated as the consumption of 1% sucrose solution/ (consumption of 1% sucrose solution + consumption of distilled water) × 100%.

**The tail suspension test (TST):** In this test, the tails of the mice were fixed at about 1/3 to the tail end with paper tape, hanging the mice on a stand 15 cm away from the surface of a table. After the tails were fixed firmly, the movement of the mice was recorded by video, with a background that had a color clearly contrasting with the hair color of the mice. The entire recording process lasted for 6 minutes. An animal behavior analysis software was used to record the immobility time of mice over the last 4 minutes (the immobility referred to the mice being hanged their motionlessly). The result showed that the immobility time of mice was positively correlated with the severity of depression-like behaviors[4,5].

**The forced swimming test (FST)**: The FST was used to evaluate the depressive-like behaviors of mice[6,7]. The experiment was performed in a transparent experiment bucket with clean water (depth: 30 cm, water temperature: 25 ± 2 ℃). The test lasted for 2 days. On the 1st day, the mice were forced to participate in adaptive swimming training for 15 minutes, and at the same time on the next day, the mice were put into the experimental barrel under the same conditions. The Motor-Monitor system was used for video recording. The test lasted for 6 minutes: the first 2 minutes was the adaption phase, followed by the recording of the immobility time of the mice in the water over the last 4 minutes (immobility in this test referred to the mice losing the desire for survival, with the judgment standard being that the body was in a state of suspension without twisting and the limbs did not move or only the hind limbs moved slightly). The immobility of mice during FST could be used as an evaluation index for depression symptoms in animal models.

**The fear conditioning (FC) training test:** This is a test to evaluate the negative emotional memory of mice[8]. There were 3 sessions of beep-shock training in a shock box. In each training session, a 30-second beep sound was played as a conditioned stimulus, and a 1-second foot shock was given as an unconditioned stimulus at the 29th second of the beep sound; the interval between two training sessions was 30 seconds. The immobility time within 2 minutes was recorded.

**References**

1. Bertola A, Mathews S, Ki SH, Wang H, Gao B (2013) Mouse model of chronic and binge ethanol feeding (the NIAAA model). Nature protocols 8 (3):627-637

2. Hall CS (1934) Drive and emotionality: factors associated with adjustment in the rat. Journal of Comparative Psychology 17 (1):89

3. Kennett GA, Chaouloff F, Marcou M, Curzon G (1986) Female rats are more vulnerable than males in an animal model of depression: the possible role of serotonin. Brain research 382 (2):416-421

4. Can A, Dao DT, Terrillion CE, Piantadosi SC, Bhat S, Gould TD (2012) The tail suspension test. JoVE (Journal of Visualized Experiments) (59):e3769

5. Hiraoka K, Motomura K, Yanagida S, Ohashi A, Ishisaka-Furuno N, Kanba S (2017) Pattern of c-Fos expression induced by tail suspension test in the mouse brain. Heliyon 3 (6):e00316

6. Petit-Demouliere B, Chenu F, Bourin M (2005) Forced swimming test in mice: a review of antidepressant activity. Psychopharmacology 177 (3):245-255

7. Yankelevitch-Yahav R, Franko M, Huly A, Doron R (2015) The forced swim test as a model of depressive-like behavior. JoVE (Journal of Visualized Experiments) (97):e52587

8. Campos AC, Fogaça MV, Aguiar DC, Guimarães FS (2013) Animal models of anxiety disorders and stress. Revista brasileira de psiquiatria (Sao Paulo, Brazil : 1999) 35 Suppl 2:S101-111. doi:10.1590/1516-4446-2013-1139

**Supplemental Figures**

**
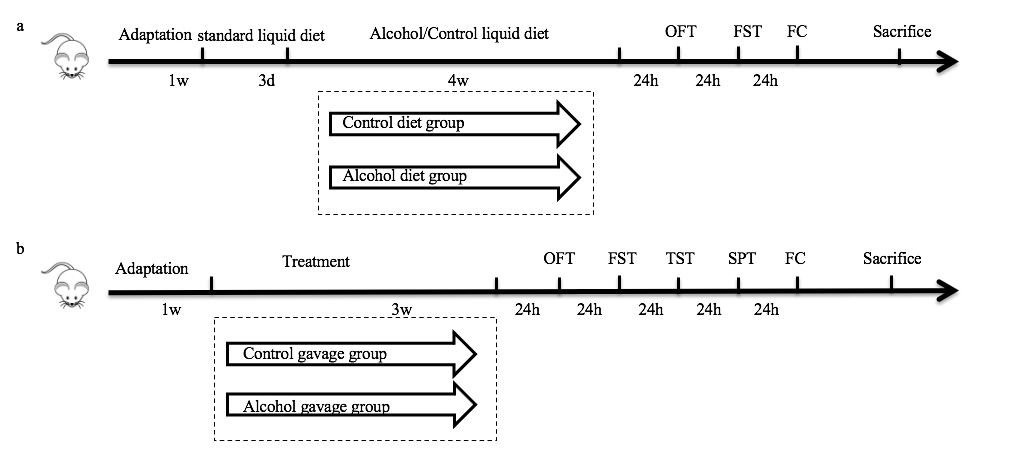
**

Supplementary Figure 1. The establishment of mouse model of alcohol induced depressive-like behaviors. (a) The timeline for establishment of the NIAAA model. (b) The timeline of establishment of the chronic alcohol gavage model.


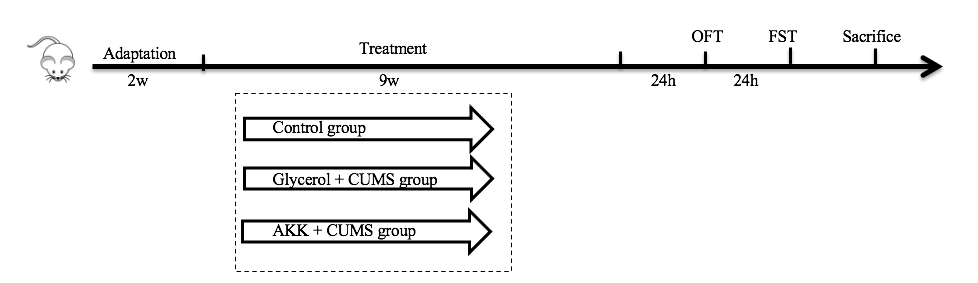


Supplementary Figure 2. The procedure of establishment of the CUMS model and the timeline of AKK treatment for CUMS mice.
